# Development of a blood proteins-based model for bronchopulmonary dysplasia prediction in premature infants

**DOI:** 10.1186/s12887-023-04065-3

**Published:** 2023-06-17

**Authors:** Wanting Ou, KeJing Lei, Huanhuan Wang, Hongmei Ma, Xiaojuan Deng, Pengcheng He, Liping Zhao, Youdao Lv, Guohong Tang, Benjin Zhang, Jie Li

**Affiliations:** 1 Department of Pediatrics, Dazhou Central Hospital, Dazhou, Sichuan China; 2 Department of Clinical Research Center, Dazhou Central Hospital, Dazhou, Sichuan China

**Keywords:** Bronchopulmonary dysplasia, Proteins model, WGCNA, LASSO, Premature infants

## Abstract

**Background:**

Bronchopulmonary dysplasia (BPD) is the most common chronic pulmonary disease in premature infants. Blood proteins may be early predictors of the development of this disease.

**Methods:**

In this study, protein expression profiles (blood samples during their first week of life) and clinical data of the GSE121097 was downloaded from the Gene Expression Omnibus. Weighted gene co-expression network analysis (WGCNA) and differential protein analysis were carried out for variable dimensionality reduction and feature selection. Least absolute shrinkage and selection operator (LASSO) were conducted for BPD prediction model development. The performance of the model was evaluated by the receiver operating characteristic (ROC) curve, calibration curve, and decision curve.

**Results:**

The results showed that black module, magenta module and turquoise module, which included 270 proteins, were significantly correlated with the occurrence of BPD. 59 proteins overlapped between differential analysis results and above three modules. These proteins were significantly enriched in 253 GO terms and 11 KEGG signaling pathways. Then, 59 proteins were reduced to 8 proteins by LASSO analysis in the training cohort. The proteins model showed good BPD predictive performance, with an AUC of 1.00 (95% CI 0.99-1.00) and 0.96 (95% CI 0.90-1.00) in training cohort and test cohort, respectively.

**Conclusion:**

Our study established a reliable blood-protein based model for early prediction of BPD in premature infants. This may help elucidate pathways to target in lessening the burden or severity of BPD.

## Introduction

Bronchopulmonary dysplasia (BPD), a chronic pulmonary disease, is the most common factor affecting mortality and long-term morbidity in premature infants [1]. In recent years, with the application of prenatal glucocorticoids, postnatal alveolar surfactant (PS) replacement and early protective ventilation strategies, the mortality rate of premature infants has decreased significantly, but the incidence of BPD has shown an upward trend as the youngest and sickest patients are now surviving[2]. Surviving children are prone to respiratory and non-respiratory comorbidities, and the long-term effects can continue to childhood, adolescence or even adulthood, which seriously endangers the health and quality of life of premature infants [3, 4]. BPD is a major challenge for neonatal intensive care unit in China, and its related long-term complications will become a serious disease burden in China. Therefore, identifying early predictors can lead to the development of targeted therapies that may reduce the neonatal and infant mortality rate and lessen the disease burden that may last through childhood and into adulthood.

The diagnosis of BPD is mainly based on preterm oxygen dependence [5]. The diagnosis is possibly made at 28 days, but may be up to 3 months after birth depending on what gestational age the patient was born. Due to the continuity and complexity of pathogenic factors and the particularity of diagnostic criteria, its special clinical manifestations are often lagging behind, resulting in the difficulty of early diagnosis [6]. Therefore, early prediction and timely intervention would be greatly beneficial as this could assist in targeting specific therapies, thereby reducing the risk of developing BPD or reducing the short- and long-term burden of disease. In addition, the mechanism of hyperoxia-induced lung injury in premature infants is closely related to the imbalance of oxidative stress response, excessive activation of cytokines, nitric oxide, neutrophils, and changes in alveolar surfactant [6, 7]. The changes of these specific protein markers already exist in the body at the early stage of BPD, which makes it possible to explore the early prediction of BPD from the perspective of proteomics.

In this study, we aimed to develop a model based on protein expression from the blood samples during their first week of life to predict the occurrence of BPD in premature infants. The predictive model could help clinicians provide timely intervention and choose appropriate therapeutic approaches for premature infants with a high risk of BPD.

## Materials and methods

### Data collection and preprocessing

**Fig. 1:**
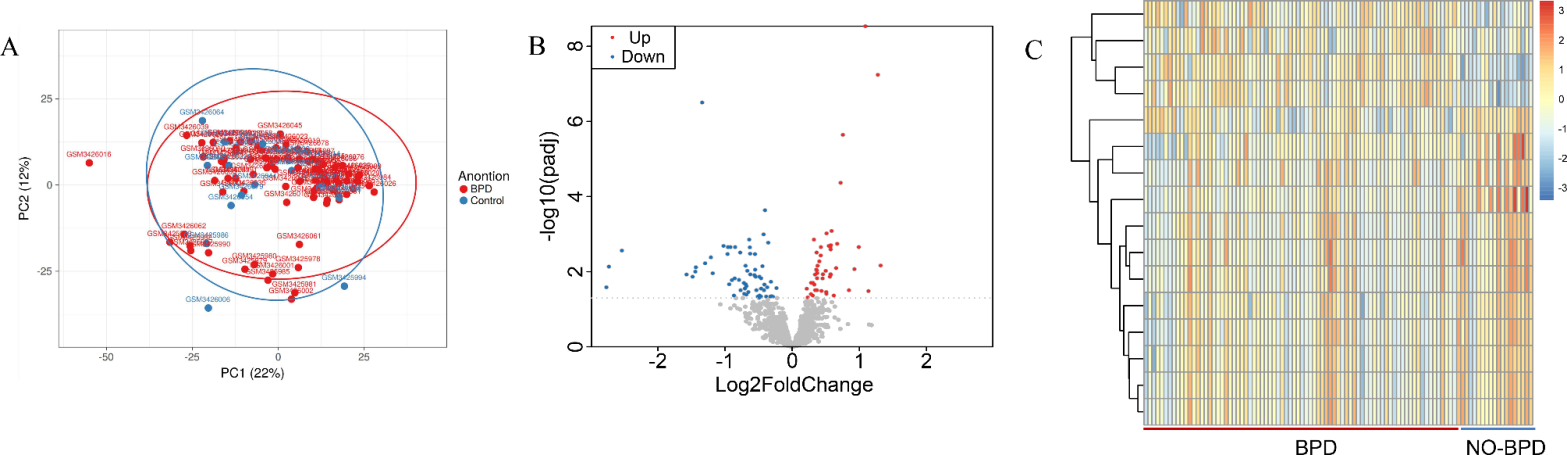
Outlier samples screen and differentially expressed proteins (DEPs) identification (**A**) Principal components analysis of patients. (**B**) Volcano plot of DEPs. (**C**) Heatmap of DEPs.

The expression profile data GSE121097 was downloaded from the Gene Expression Omnibus (GEO) website of the National Center for Biotechnology Information (NCBI) in the United States (https://www.ncbi.nlm.nih.gov/geo/). The data set contained 102 samples including 20 patients without BPD (NO-BPD group) and 82 patients with BPD (BPD group). The enrolled infants had a gestational age less than 34 weeks at birth and a birthweight between 500 and 1250 g. The average (± standard deviation) gestation age was 29.10 ± 1.52 weeks in NO-BPD group (n = 20), 25.84 ± 1.66 weeks in BPD group (n = 82). And 102 blood samples from infants were collected at 7 days (± 48 h) after birth with EDTA-plasma tube. The proteomics detection from blood samples was based on SOMAscanTM technology. All the protein expression values were normalized by “log_2_(expression + 1)” transformation. Principal Component analysis (PCA) was used to remove abnormal samples from the NO-BPD group and BPD group, with 2 samples in NO-BPD group and 3 samples in BPD group removed (Fig. 1A), and the remaining 97 samples (18 samples in NO-BPD group and 79 samples in BPD group) included in final analysis.

### Definition of BPD

BPD status was assessed at 36 weeks postmenstrual age (PMA) according to a modification of the NIH Workshop definition [8] that included application of the standardized oxygen reduction test [9]. Detailed diagnostic criteria of BPD status were as follows [10]: Infants who received < 28 days oxygen and were on room air at 36 weeks PMA were assigned to the outcome of No BPD. Infants on positive pressure support or receiving ≥ 30% supplemental oxygen at 36 weeks PMA were assigned the outcome severe BPD. Infants receiving > 28 days of supplemental oxygen but were on room at 36 weeks PMA were assigned to the outcome of Mild BPD. Those receiving < 30% supplemental oxygen at 36 weeks PMA underwent oxygen reduction testing to differentiate between No BPD (required < 28 days of oxygen and passed reduction test), Mild BPD (required > 28 days of oxygen and passed oxygen reduction test), or Moderate BPD (required > 28 days of oxygen and did not pass the reduction test). Infants diagnosed with BPD were assigned to the BPD group, and infants diagnosed without BPD were assigned to the NO-BPD group.

###  Weighted gene co-expression network analysis (WGCNA)

The “limma” package of R software was used to screen the differentially expressed proteins (DEPs) between BPD and NO-BPD groups. The gene co-expression network analysis was constructed using the “WGCNA” package. In order to ensure compliance with scale-free network distribution, WGCNA needs to select an appropriate weighting coefficient β. The “pickSoftThreshold” function in WGCNA package was used to calculate the correlation coefficient of β value and the mean value of gene connectivity, and to select an appropriate soft threshold β to make the constructed network more in line with the scale-free network standard. A one-step method was used to construct the related gene network, the adjacency matrix was transformed into a topological overlap matrix TOM, and hierarchical clustering was used to generate a hierarchical clustering tree of genes. Gene significance (GS) and module membership (MM) were calculated to measure the significance of genes and clinical information, and to analyze significant associations between modules and models.

### Gene Ontology (GO) and Kyoto Encyclopedia of genes and genomes (KEGG) pathway analysis

The Search Tool for the Retrieval of Interacting Genes (STRING; http://string-db.org) (version 11.5) online database was used to perform the GO analysis, mainly including biological process, cellular component, and molecular function levels, and KEGG pathway [11, 12] enrichment analysis of the molecules in meaningful modules. The threshold false discovery rate < 0.05 was considered statistically significant.

### Development and validation of the prediction model

All participants were randomly divided into training cohort and test cohort at a ratio of 7:3. The least absolute shrinkage and selection operator (LASSO) method followed by 5-fold cross-validation was applied to select the most useful predictive proteins for BPD prediction in the training cohort. The receiver operating characteristic (ROC) curve, calibration curve and decision curve were evaluated for the performance of the model. The optimal critical threshold was determined by the Youden Index.

## Results

### DEPs associated with BPD

In total, we identified 108 DEPs between NO-BPD group and BPD group based on the criteria p-adj < 0.05, of which 47 were upregulated proteins and 61 were downregulated proteins in BPD patients. Heatmap and volcano plot of those DEPs are shown in Fig. 1B C.

**Fig. 2:**
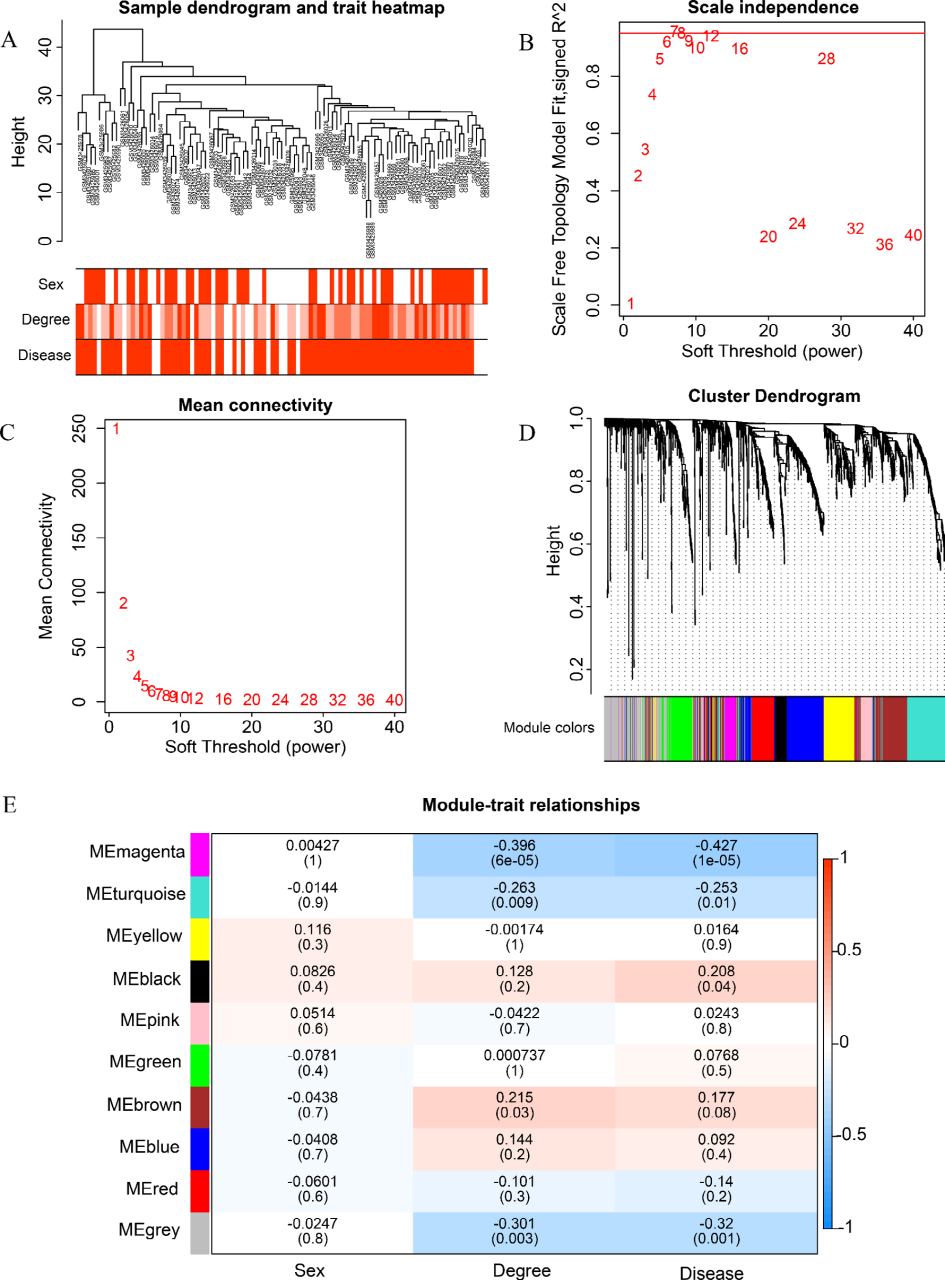
Weighted gene co-expression network analysis (WGCNA) (**A**) Dendrogram of the remaining 97 samples. (**B**) The scale-free fit index (y-axis) as a function of the soft-thresholding power (x-axis). (**C**) Network connectivity under different soft thresholds. (**D**) Clustering dendrograms of proteins, with dissimilarity based on topological overlap, together with assigned module colors. (**E**) Module-trait associations

Further, a hierarchical cluster map of 97 samples was drawn by calculating the correlation between samples, and no abnormal samples were deleted here. These samples were used for subsequent WGCNA module construction (Fig. 2A). The scale-free network was established to screen out gene modules highly related to BPD, and the scale independence and average connectivity of the weighted co-expression network were determined. The soft threshold β was selected as 10 (scale-free R^2^ = 0.95), and the modules were finally determined (Fig. 2B and D, non-clustering modules were gray). A total of 10 modules were identified in this study, and black modules (r = 0.208, *p* = 0.04) were positively correlated with BPD while magenta (r = -0.427, *p* = 1e-05) and turquoise (r = -0.253, *p* = 0.01) modules were negatively correlated with BPD (Fig. 2E), thus selected as vital modules for further study.

### Function annotation of significant proteins

**Fig. 3:**
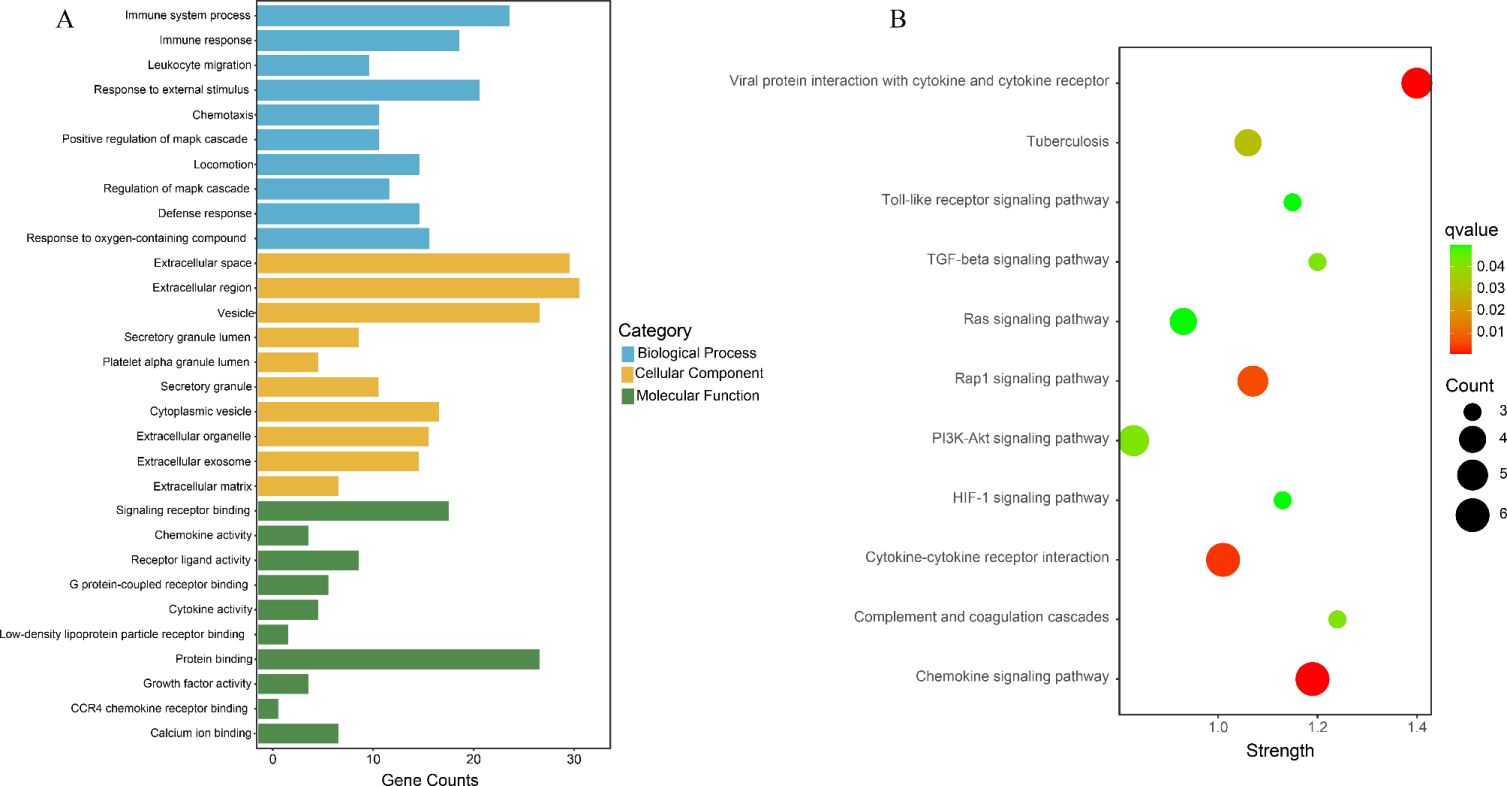
Functional analysis of the identified proteins (**A**) The top 10 Gene ontology (GO) terms in each category of biological process, cellular component and molecular function. (**B**) Kyoto Encyclopedia of Genes and Genomes (KEGG) pathways of the identified proteins

In total, 270 proteins were included in the three modules (black module: 57 proteins; magenta module: 43 proteins; turquoise: 170 proteins). 59 proteins were overlapped between “limma” analysis and the three modules. We further performed GO and KEGG enrichment analysis in the STRING online database. According to the functional enrichment results, 221 biological process terms, 18 cellular component terms and 14 molecular function terms were statistically significantly enriched in these proteins. Top 10 terms of each categories based on false discovery rate value are displayed in Fig. 3A. KEGG enrichment analysis results revealed 11 significant signaling pathways (Fig. 3B), and the top four enriched pathways were Viral protein interaction with cytokine and cytokine receptor, Rap1 signaling pathway, Cytokine-cytokine receptor interaction and Chemokine signaling pathway, respectively.

### Development and validation of the proteins model

**Fig. 4:**
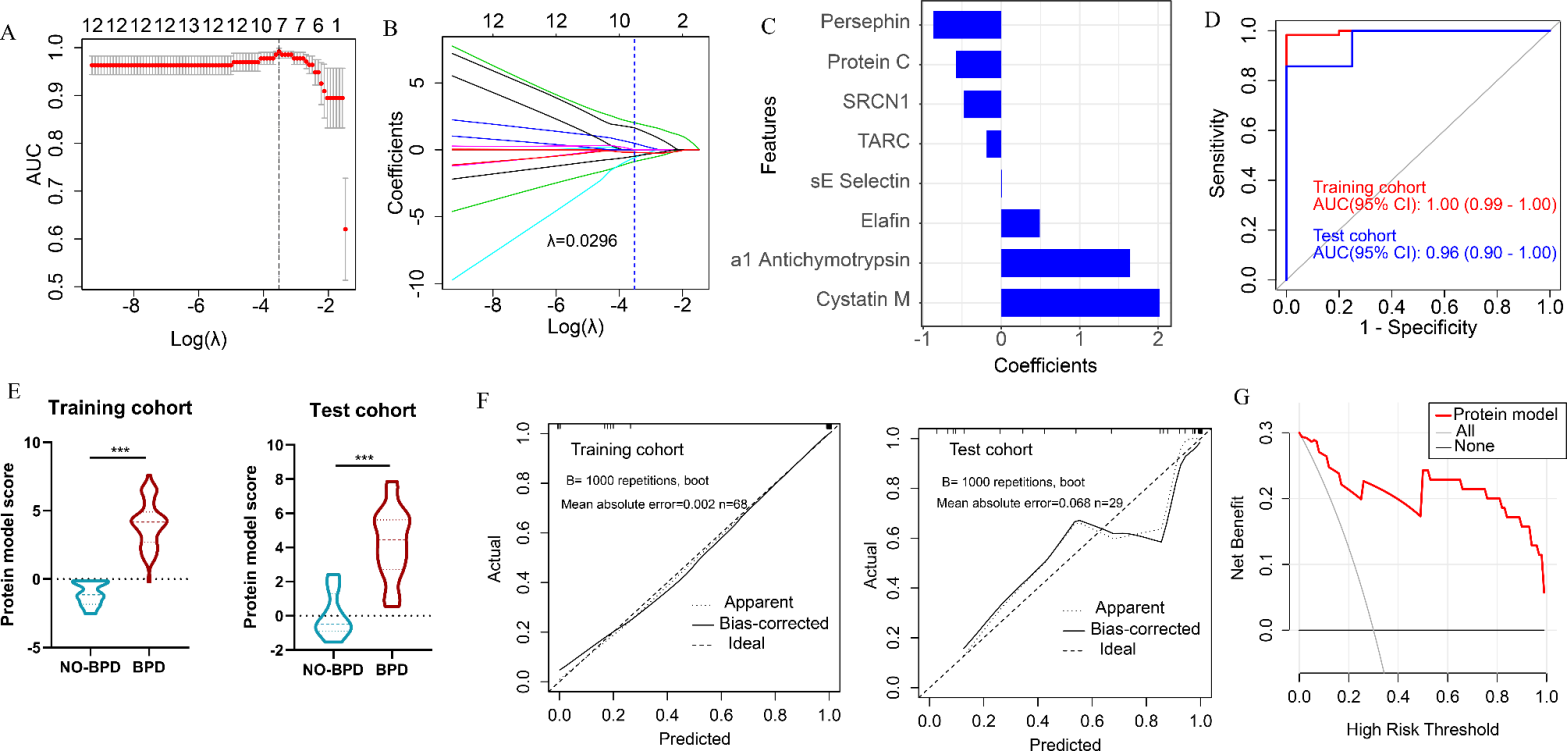
Development and validation of the proteins model (**A**) Tuning parameter (λ) selection in the least absolute shrinkage and selection operator (LASSO) used 5-fold cross-validation via minimum criteria. (**B**) LASSO coefficient profiles of the 59 proteins and the optimal λ (λ = 0.0296) resulted in 8 nonzero coefficients. (**C**) Eight proteins for model development. (**D**) The area under the receiver operating characteristic (AUC) curves of the proteins model in training and test cohorts. (**E**) Violin plot of protein score based on the proteins model between non-BPD and BPD groups in the training and test cohorts. (**F**) Calibration curves of the proteins model in the training and test cohorts. (**G**) Decision curve of the proteins model Protein model score = − 43.250 + 2.021*Cystatin M + 1.644* a1 Antichymotrypsin + 0.492*Elafin + 0.005*sE Selectin − 0.187*TARC − 0.476*SRCN1–0.581*Protein C − 0.871*Persephin

Of the 59 overlapped proteins, eight proteins were selected as predictors on the basis of 68 participants in the training cohort (Fig. 4A-C). These proteins with nonzero coefficients were presented in the protein model score calculation formula. The proteins model yielded an AUC value of 1.00 (95% CI 0.99-1.00) in the training cohort, with a sensitivity of 0.98 (95% CI 0.95-1.00), specificity of 1.00 (95% CI 1.00–1.00). Importantly, the model achieved an AUC value of 0.96 (95% CI 0.90-1.00) in the test cohort, with a sensitivity of 0.90 (95% CI 0.71-1.00), specificity of 1.00 (95% CI 0.87-1.00) (Fig. 4D; Table 1). And the distributions of protein model score between NO-BPD group and BPD group in training and test cohorts are displayed in the Fig. 4E. Significant difference was observed between NO-BPD and BPD groups in both cohorts. And the optimal cut-off score of the proteins model for BPD prediction calculated in training cohort was 0.579.

Calibration curve suggested good consistency between prediction and observation of the predictive model in the training and test cohorts (Fig. 4F). The decision curve demonstrated that using the proteins model to predict BPD could increase more net benefit than either the all items or the none item (Fig. 4G).

**Table 1:** Prediction Performance of the Proteins Model in Training and Test Cohorts

Items	Training cohort	Test cohort
AUC	1.00 (0.99−1.00)	0.96 (0.90−1.00)
Sensitivity	0.98 (0.95−1.00)	0.90 (0.71−1.00)
Specificity	1.00 (1.00–1.00)	1.00 (0.87−1.00)
Accuracy	0.98 (0.96−1.00)	0.93 (0.79−1.00)
NPV	0.91 (0.77−1.00)	0.80 (0.57−1.00)
PPV	1.00 (1.00–1.00)	1.00 (0.95−1.00)

AUC, Area under the ROC curve; NPV, Negative Predictive Value; PPV, Positive Predictive Value

## Discussion

With the deepening of scholars’ research on BPD, it is found that the pathogenesis of BPD involves the complex interaction between genetic factors and environmental factors, which is based on genetic susceptibility by hyperoxia, mechanical ventilation, intrauterine and postnatal inflammation, and other factors. Previous study has confirmed that the non-synonymous gene mutation may be related to the occurrence of BPD by sequencing the gene exons of 100 children with BPD and the control group [13]. Also, recent studies have highlighted the role of mRNA, non-coding RNA, proteins, microbiome and metabolites in BPD through omics approaches [14]. As proteins are the direct embodiment of life phenomena, the study of protein structure and function should clarify the mechanism of life changes under physiologic or pathological conditions. Therefore, exploring the crucial proteins and related-pathways are important to understand the pathogenic mechanisms and further develop novel therapeutic strategies of BPD.

Previous studies have shown that the development of BPD is characterized by inflammation, extracellular matrix remodeling and apoptosis, and is closely related to the disorder of growth factor signal transduction [15]. Recent studies have also confirmed the role of transforming growth factor-β (TGF-β) in BPD development, by promoting fibrosis of lung cell [16, 17]. A large number of animal studies have shown that increased TGF-β level can be observed in animal models of BPD induced by hyperoxia exposure and prenatal inflammation, and obvious fibrosis and inhibited alveolar development can be seen in the early postnatal period (7–14 days) [18–21]. In contrast, inhibiting TGF-β with neutralizing antibody alleviates these changes seen in hyperoxic BPD [18, 19]. Pathways enrichment analysis based on differentially expressed proteins demonstrated the TGF-β signaling pathway regulated by DEPs in BPD patients, which is consistent with the conclusion of other studies that the TGF-β signaling pathway plays an important role in the development of BPD.

Animal and in vitro studies found that the protective effect of FGF-7 decreased significantly after gene silencing inhibition of AKT [22, 23]. In addition, in vitro experiments found that PI3K inhibitor could counteract the cell proliferation induced by FGF-7, accompanied by a decrease in PI3K and AKT level [24]. Therefore, it is speculated that PI3K-Akt signaling pathway may be involved in the occurrence and development of BPD. In the present study, pathways analysis based on protein levels among patients showed PI3K-Akt signaling pathway was significantly altered in BPD. Additionally, HIF-1 signaling pathway, Cytokine-cytokine receptor interaction, Rap1 signaling pathway, Ras signaling pathway and Toll-like receptor signaling pathway differed significantly between BPD and NO-BPD groups, which suggested that these biological function changes played an important role in the occurrence and development of BPD. In subsequent BPD studies, we should focus on these pathways.

WGCNA is a system biological analysis method used in genomics, proteomics and metabolomics to describe the expression correlation of corresponding genes, proteins, or metabolites, which can cluster molecules with similar expression patterns and analyze the association between modules and specific shapes or phenotypes [25]. Therefore, it is widely used in the study of disease and other traits and molecular correlation analysis [26–28]. Here we combined WGCNA and DEPs screen method for data dimension reduction and hub proteins identification. And 59 hub proteins were found, which indicated these proteins were involved in the evolution of BPD. Current diagnostic approaches mainly based on clinical definitions, imaging modalities, and biomarker data are limited by subjectivity, high radiation exposure, and patient cooperation [5]. Interestingly, blood-based biomarker tests are cost-effective and can be assayed easily and quickly with minimal sample volume. Thus, they have the potential to greatly aid the disease diagnostics.

In the present study, we selected 8 predictors for BPD prediction through LASSO regression, a method which has been widely used in feature selection and model development [29, 30]. Our model could achieve an of AUC (95% CI) 1.00 (0.99-1.00) and 0.96 (0.90-1.00) in training cohort and test cohort, respectively. These results suggested that these proteins have important value in BPD development. Finally, we identified 8 proteins in BPD patients, including Cystatin M, a1 Antichymotrypsin, Elafin, sE Selectin, TARC, SRCN1, Protein C and Persephin. Studies have characterized the protective role of activated Protein C in anti-inflammatory, anti-apoptotic activities and endothelial barrier stabilization [31]. Ina Rudloff et al. have found that Protein C could reduce the lung structural damage induced by BPD, with significant effect in IL-1b, IL-1Ra, IL-6 decrease [32]. Elafin is a serine protease inhibitor that could bind to extracellular matrix (ECM) proteins thereby protecting against injury induced by sustained inflammation [33]. Animal study has found that the Elastin gene-deficient mice die soon after birth from developmental defects including loss of alveolar separation and airway branching [34, 35]. Wenli Han et al. have reported Elafin could inhibit elastase and activation of the TGF-β1 signaling cascade, hence ameliorating apoptosis, inflammation and the elastin organization in the alveoli based on O^2^-induced lung injury model in neonates [36]. Elevated serum levels of sE-selectin have been found in a variety of other inflammatory conditions including asthma and septic shock, which was closely associated with disease severity and outcome [37–39]. Also, study has revealed that higher cord blood levels of sE-selectin in the tracheal aspirate at birth are associated with increased risk of BPD development in preterm infants [40]. These results suggested that the protein model composed of these proteins for BPD prediction was interpretable.

In our work, some limitations need to be acknowledged. First, the gestational age differed significantly in the no-BPD and BPD groups, thus the effect of time on the expression of proteins could affect the generalizability. Thus, the robustness and generalizability of the eight-protein model requires further optimization and validation in large-scale prospective multi-center cohorts. Secondly, although experimental data of the correlation between some of the proteins identified in this paper and BPD have been reported in academic literature, more evidence is needed to elucidate the inherent correlation and specific function mechanism between the eight-protein and the occurrence and development of BPD. Despite these drawbacks, our results demonstrated valuable information on the importance and significance of the eight-protein model in early prediction and evaluating BPD in high risk premature infants.

In conclusion, our study revealed significantly altered and hub proteins in the blood of premature infants with BPD through WGCNA and differential protein analysis, and identified significantly enriched pathways in the disease, which could provide potential targets for the treatment of BPD and novel clues for understanding the pathogenesis of the disease. As such, we proposed a blood-based reliable model for BPD prediction, which could efficiently and conveniently predict and evaluate BPD in high risk premature infants and further provide pathways to target to prevent or lessen the severity of BPD.

## Data Availability

The datasets analyzed during the current study are available in the GEO database (record, GSE121097) [https://www.ncbi.nlm.nih.gov/geo/query/acc.cgi?acc=GSE121097].
